# Synergistic Effects of the Combination of Alpelisib (PI3K Inhibitor) and Ribociclib (CDK4/6 Inhibitor) in Preclinical Colorectal Cancer Models

**DOI:** 10.3390/ijms252413264

**Published:** 2024-12-10

**Authors:** Razia Aslam, Cathy E. Richards, Joanna Fay, Lance Hudson, Julie Workman, Cha Len Lee, Adrian Murphy, Brian O’Neill, Sinead Toomey, Bryan T. Hennessy

**Affiliations:** 1 Medical Oncology Group, Department of Medicine, RCSI University of Medicine and Health Sciences, D09 YD60 Dublin, Ireland; raslam33@gmail.com (R.A.); catherinerichards@rcsi.ie (C.E.R.);; 2 Beaumont RCSI Cancer Centre, D09 YD60 Dublin, Ireland; 3 Department of Medical Oncology, St James’s Hospital, D08 NHY1 Dublin, Ireland; 4 RCSI Biobank, RCSI University of Medicine and Health Sciences, D09 YD60 Dublin, Ireland; 5 Department of Pathology, RCSI University of Medicine and Health Science, D09 YD60 Dublin, Ireland; 6 Department of Surgery, RCSI University of Medicine and Health Science, D09 YD60 Dublin, Ireland; lhudson@rcsi.ie; 7 Department of Medical Oncology, Beaumont Hospital, D09 YD60 Dublin, Ireland; 8 Department of Radiation Oncology, St. Luke’s Radiation Oncology Centre, Beaumont Hospital, D09 YD60 Dublin, Ireland

**Keywords:** colorectal cancer, drug combinations, alpelisib, ribociclib, targeted therapies

## Abstract

The CDK4/6 inhibitor Ribociclib has shown limited efficacy as a monotherapy in colorectal cancer (CRC). However, combining Ribociclib with targeted therapies could present a viable strategy for treating CRC. This study evaluated the combination of Ribociclib and the PI3K inhibitor Alpelisib across four distinct cell lines representing different mutational statuses (*PIK3CA/KRAS* wild-type, *KRAS*-mutated, *PIK3CA*-mutated, and *PIK3CA/KRAS*-mutated). We analyzed the drugs’ impact on key proteins involved in the PI3K pathway, cell cycle regulation, and apoptosis. The combination of Alpelisib and Ribociclib demonstrated a synergistic anti-proliferative effect across all cell lines, leading to a simultaneous decrease in pRB, pAKT, and p-S6 levels, and a more comprehensive suppression of the PI3K/AKT/mTOR pathway. Additionally, there was an upregulation of the apoptotic marker, p-BCL2, in cells treated with the combination compared to controls. In vivo studies using Caco-2, LS1034, and SNUC4 xenografts revealed a significant reduction in tumour growth with the combination therapy compared to single-agent treatments. These findings suggest that combining Alpelisib and Ribociclib could be a promising therapeutic approach for CRC, warranting further clinical exploration.

## 1. Introduction

Colorectal cancer (CRC) ranks as the third most prevalent cancer worldwide and the second leading cause of cancer-related deaths after lung cancer [1]. Data from the US National Cancer Institute’s SEER database shows that patients with distant metastases have a five-year survival rate of less than 15%, while those with localized disease have a much better prognosis, with a five-year survival rate of around 70–90% [2]. Risk factors for colorectal cancer include both lifestyle and genetic factors. Modifiable risks include diets high in red or processed meats, low physical activity, obesity, smoking, and excessive alcohol use. Family history, genetic conditions like Lynch syndrome or Familial adenomatous polyposis (FAP), and inflammatory bowel diseases also increase susceptibility. Emerging evidence suggests gut microbiota changes, driven by antibiotic use or diet, may also play a role. The global burden of CRC is projected to rise by 60% by 2030 [3]. The precise mechanisms contributing to the increase in the incidence of colorectal cancer are likely due to a combination of lifestyle and environmental changes. Diets which are increasingly rich in processed and ultra-processed foods, coupled with higher rates of obesity, are likely to play a significant role, as are alterations in gut microbiota potentially driven by widespread antibiotic use and dietary patterns. In addition, sedentary lifestyles and reduced physical activity may also be important. The protective factors that can help reduce the risk of developing colorectal cancer include a diet high in fibre, fruits, vegetables and whole grains, as well as regular physical activity and maintaining a healthy weight. Limiting red and processed meats and avoiding smoking and excessive alcohol consumption also reduce the risks. Routine screening can identify and remove precancerous polyps. Some studies suggest that aspirin use and vitamin D sufficiency may offer additional protective effects [1].

CRC pathology and prognosis are influenced by various histological, molecular, and clinical factors. Histologically, most CRCs are adenocarcinomas and are graded by glandular differentiation. Poorly differentiated or mucinous subtypes generally have a worse prognosis. Immunohistochemistry (IHC) can identify mismatch repair (MMR) protein deficiencies, indicating microsatellite instability (MSI), which is associated with better outcomes and responses to immune checkpoint inhibitors. Prognosis is also guided by the TNM staging system, with tumour depth (T), nodal involvement (N), and distant metastases (M) being important factors. Local tumour extent, lymphovascular invasion, and perineural invasion signal advanced disease and poorer outcomes. Extramural venous invasion, particularly into larger veins, correlates with higher metastatic risk. Molecular profiling can identify actionable mutations, such as RAS and BRAF, which can help predict responders and non-responders to specific targeted therapies [4].

Treatment for CRC is multidisciplinary and depends on the stage and molecular characteristics of the disease. Surgical resection is the mainstay of treatment for localized CRC, with complete mesocolic or total mesorectal excision to remove the tumour and associated lymph nodes. For rectal cancer, radiotherapy is particularly effective and neoadjuvant chemoradiotherapy is often used to shrink tumours before surgery and reduce recurrence risk. Advanced or metastatic CRC may involve systemic chemotherapy, often combined with targeted agents, while microsatellite instability-high (MSI-H) or mismatch repair-deficient (dMMR) tumours are increasingly treated with checkpoint inhibitors [1]. Fluorouracil (5-FU) is a standard first-line chemotherapy for colorectal cancers, particularly in distal colon tumours [5]. Despite advancements in treatments, about 30% of patients progress to stage IV disease, with a five-year overall survival rate of less than 15% [6], highlighting the urgent need for novel therapeutic strategies for metastatic CRC. For Stage IV CRC, regimens like FOLFOX, FOLFIRI, and FOLFOXIRI are often combined with monoclonal antibodies targeting EGFR or VEGF, such as cetuximab and bevacizumab, which have extended overall survival (OS) to over 24 months [7]. However, despite initial successes, most patients eventually relapse, with later treatment lines offering limited clinical benefit, highlighting the urgent need for novel therapeutic strategies for metastatic CRC.

Drug resistance in colorectal cancer (CRC) often develops within 6–12 months of treatment, reducing the clinical efficacy of anticancer therapies. This resistance arises from mechanisms such as secondary mutations, which contribute to tumour heterogeneity [8], and the activation of alternative signalling pathways, including MAPK/ERK and PI3K/AKT/mTOR [9,10,11].

The MAPK and PI3K pathways are among the most frequently activated signalling pathways in human cancers, including CRC, and are closely interconnected [11,12]. The MAPK/ERK pathway (RAS/RAF/MEK/ERK) regulates key cellular processes such as growth, survival, and proliferation [13], while the PI3K/AKT/mTOR pathway promotes cell progression, angiogenesis, and invasive potential [14]. Both pathways are involved in the development of drug resistance, and feedback loops enable one pathway to activate or inhibit the other [15,16]. Our previous research has demonstrated that somatic genetic alterations activating the PI3K and MAPK signalling pathways are prevalent in rectal cancer and are associated with resistance to chemotherapy and radiotherapy, leading to poor patient outcomes [17]. However, combined therapies targeting multiple pathways show promise in overcoming this resistance [18,19]. Cyclin-dependent kinases (CDKs) play a role in cell cycle regulation, and CDK4/6 inhibitors have emerged as therapeutic targets and shown anticancer activity in CRC [20]. There is a strong scientific rationale for combining CDK4/6 inhibitors with PI3K-targeting agents [21]. Studies in breast cancer models have shown that combining CDK4/6 and PI3K inhibitors can prevent resistance to PI3K inhibitors while also stopping cancer cells from adapting to CDK4/6 inhibitors [22,23,24,25].

When CDK4/6 inhibitors (Palbociclib, Abemaciclib, and Ribociclib) were introduced to the clinic, they represented a real breakthrough for the treatment of HR+/HER2- luminal BC patients. The addition of a CDK4/6 inhibitor to the standard endocrine therapy showed impressive results, extending median progression-free survival and prolonging median overall survival of advanced/metastatic luminal BC patients [26]. Recent preclinical studies have explored the use of CDK4/6 inhibitors in combination with other non-myelosuppressive agents in CRC, showing the potential to delay resistance or enhance responses during disease progression [26].

In recent years, large-scale genomic sequencing studies have provided insight into the molecular landscape of CRC [27,28]. Key mutations frequently found in CRC include *KRAS*, *PIK3CA*, *BRAF*, *APC*, and *TP53*, many of which activate the MAPK and PI3K pathways and are, thus, considered to be potentially druggable. *KRAS* mutations are common in CRC, occurring in 40–65% of tumours and are known to cause resistance to EGFR-targeted therapies [29]. While *KRAS* G12C mutations have shown promise for targeted treatment with the *KRAS* inhibitor Sotorasib in lung cancer, resistance to the therapy develops rapidly [30], and there are currently no clinically approved inhibitors available to target *KRAS* G12D, the most frequent *KRAS* mutation identified in CRCs. *PIK3CA* mutations are found in up to 25% of CRCs, and mutations in exons 9 and 20 play a significant role in therapy resistance, particularly to EGFR-targeted and chemotherapy treatments [31]. *PIK3CA* mutations often co-occur with *KRAS* or *BRAF* mutations [32], complicating treatment strategies.

This study investigates the combination of two targeted agents, Ribociclib (a CDK4/6 inhibitor) and Alpelisib (a PI3K inhibitor), as a novel treatment strategy aimed at overcoming drug resistance in CRC. Although both drugs have shown some single-agent activity in colorectal cancer (CRC), early trials did not demonstrate significant anti-tumour effects when used alone [33,34]. Sensitivity to CDK4/6 inhibitors may be associated with genetic factors such as co-deletion of CDKN2A/CDKN2C [35]. However, the drug primarily induces cytostatic effects, not cytotoxic, and resistance may develop via Rb-dependent or Rb-independent pathways [35]. Prior clinical studies with PI3K inhibitors in CRC have been unsuccessful due to toxicity concerns [36,37]. However, Alpelisib’s more favourable pharmacodynamic profile makes it a promising candidate for future trials [38], particularly in combination with other targeted agents. Preclinical evidence using other PI3K and CDK4/6 inhibitors suggests that the combination of Alpelisib and Ribociclib may produce synergistic benefits in specific CRC subpopulations [21]. Given the roles of the PI3K and MAPK pathways in CRC, the rationale for combining these inhibitors is based on their potential to overcome resistance and provide enhanced therapeutic benefits, especially in CRC subpopulations with relevant genetic mutations. By utilizing these two inhibitors, this study aims to investigate the combination of Ribociclib and Alpelisib as a potential therapeutic approach to justify further in vivo studies and future clinical trials in CRC patients.

## 2. Results

### 2.1. Effects of Alpelisib and Ribociclib on Cell Viability in CRC Cell Lines with Different Mutational Backgrounds

**Figure 1:**
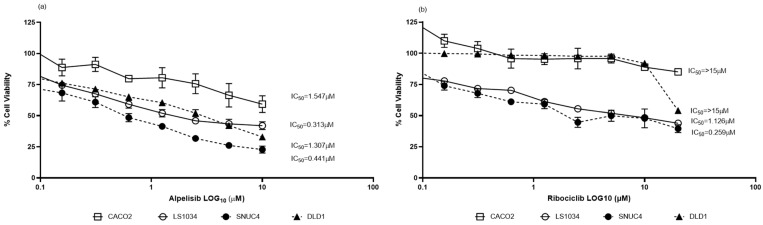
Cytotoxicity effect of (**a**) Alpelisib and (**b**) Ribociclib in Caco-2 (*PIK3CA/KRAS* wild-type), LS1034 (*KRAS* mutated), SNUC4 (*PIK3CA* mutated), and DLD-1 (*PIK3CA/KRAS* mutated) cell lines and their corresponding IC_50_ values. Cells were treated with serial 1:2 dilutions of the PI3K inhibitor Alpelisib (10 µM–0.08 µM) or the CDK4/6 inhibitor Ribociclib (20 µM–0.08 µM) or DMSO control. Cells were incubated at 37˚C with 5% CO_2_ for 7 days, and proliferation was measured using an acid phosphatase toxicity assay. Assays were run in at least three independent experiments, and data are presented as mean ± SEM. GraphPad PRISM version 9.0 was used to calculate the IC_50_ (inhibitory concentration 50) of each drug for each cell line. Assays were run in at least three independent experiments, and data are presented as mean ± SEM.

Caco-2 (*PIK3CA/KRAS* wild-type) and DLD-1 (*PIK3CA/KRAS* mutated) cell lines showed resistance to Ribociclib (IC_50_ > 15 µM for both). In contrast, LS1034 (*KRAS* mutated) and SNUC4 (*PIK3CA* mutated) were sensitive to Ribociclib, with IC_50_ values of 1.126 µM and 0.259 µM, respectively. Sensitivity to Alpelisib varied across cell lines: LS1034 (0.313 µM), SNUC4 (0.441 µM), DLD-1 (1.307 µM), and Caco-2 (1.547 µM). Caco-2 was the least sensitive to both drugs, as shown in Figure 1.

### 2.2. Synergistic Effects of Alpelisib and Ribociclib on Cell Viability

**Figure 2:**
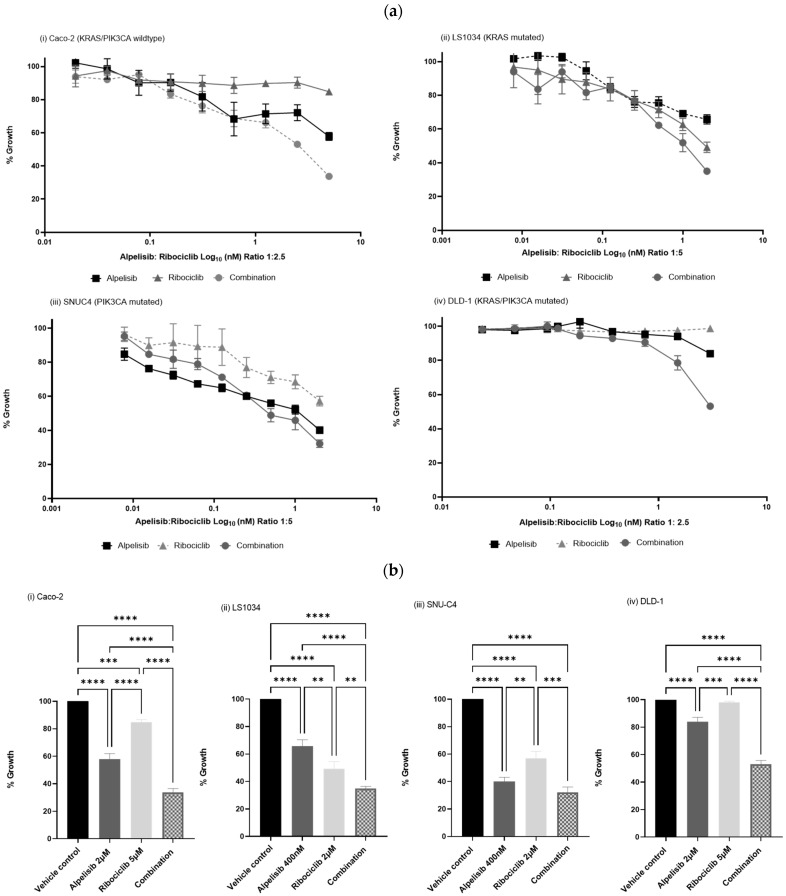
(**a**). Cell growth inhibitory effects of Alpelisib, Ribociclib, and their combination in (**i**) Caco-2 (*PIK3CA*/*KRAS* wild-type), (**ii**) LS1034 (*KRAS* mutated), (**iii**) SNU-C4 (*PIK3CA* mutated) and (**iv**) DLD-1 (*PIK3CA/KRAS* mutated) cell lines. Each cell line was treated with increasing concentrations of Ribociclib, Alpelisib, and their combination at various fixed ratio doses which were pre-determined by the single agent IC_50_ values (2 µM–0.15 µM Alpelisib and 5 µM–0.03 µM Ribociclib for Caco-2 and DLD-1 and 400 nM–3.13 nM Alpelisib and 2 µM–0.15 µM Ribociclib for LS1034 and SNUC4 cells). Cell viability was assessed using a 7-day acid phosphatase assay. The graphs show the mean cell growth ± standard error of mean (SEM) from a minimum of three repeats in each cell line. (**b**). Effect of Alpelisib, Ribociclib and their combination on cell viability in (**i**) Caco-2 (*PIK3CA/KRAS* wild-type), (**ii**) LS1034 (*KRAS* mutated), (**iii**) SNUC4 (*PIK3CA* mutated), and (**iv**) DLD-1 (*PIK3CA/KRAS* mutated) cells. Graphs show the maximum growth inhibition in 7-day acid phosphatase assays in three independent experiments. Ordinary one-way ANOVA with Tukey’s multiple comparisons tests was used to evaluate the effect of drug treatments (** *p* < 0.01, *** *p* < 0.001, **** *p* < 0.0001).

The combined treatment of Alpelisib and Ribociclib exhibited a synergistic anti-proliferative effect in all four CRC cell lines, as determined by the combination index (CI) using CalcuSyn software (version 2.0) based on the Chou–Talalay method [39] (Figure 2, Table 1). This synergy was observed across different mutational backgrounds: LS1034 (*KRAS* mutated, CI@ED75 = 0.56 ± 0.25), DLD-1 (*KRAS/PIK3CA* mutated, CI@ED75 = 0.03 ± 0.03), SNUC4 (*PIK3CA* mutated, CI@ED75 = 0.56 ± 0.33), and Caco-2 (*KRAS/PIK3CA* wild-type, CI@ED75 = 0.45 ± 0.22). The corresponding IC_50_ values for each drug and CI values at ED50, ED75, and ED90 are shown in Table 1.

### 2.3. Effects of Combining Alpelisib and Ribociclib on Signalling Pathway Activation

**Figure 3:**
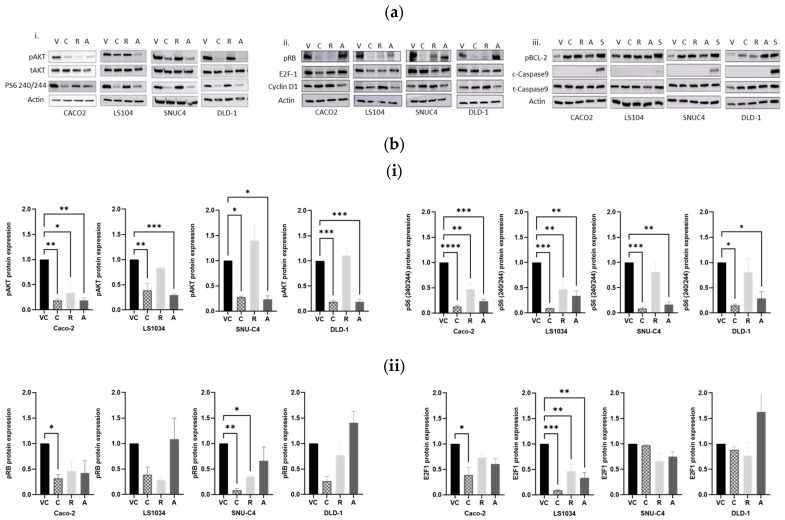

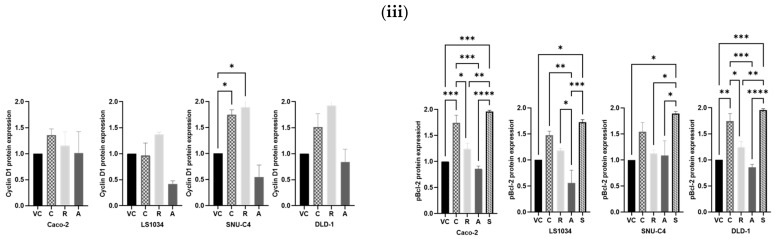
(**a**). Western blot analysis of protein expression or phosphorylation of (**i**) PI3K signalling pathway proteins, (**ii**) cell cycle proteins, and (**iii**) apoptotic proteins in colorectal cancer cell lines. Protein lysates were harvested from cells treated with Alpelisib (A), Ribociclib (R), their combination (C), and the DMSO vehicle control (VC) for 24 h for PI3K signalling pathway proteins and 72 h for all other proteins at their IC_50_ concentrations. For apoptotic proteins, staurosporine treatment (S) was used as a positive control. (**b**). Densitometry analysis of Western blots of (**i**) PI3K signalling pathway proteins, (**ii**) cell cycle proteins, and (**iii**) apoptotic proteins using Image J software. Intensities of phosphorylated proteins were normalized to the intensity of the total protein or β-actin. All *p*-values were generated using ordinary one-way ANOVA with Tukey’s multiple comparisons tests. (* *p* < 0.05, ** *p* < 0.01, *** *p* < 0.001, **** *p* < 0.0001). Data are presented as ± SEM and are representative of independent triplicate experiments. (pAKT, pS6, etc. indicates phospho AKT, phosphor S6, etc.).

Treatment with the combination of Alpelisib and Ribociclib led to a marked reduction in pAKT levels across all cell lines compared to the vehicle control (Figure 3), with the most significant decrease in DLD-1 (mean difference = 0.8148; 95% CI = 0.4619 to 1.168; *p* = 0.0004). Interestingly, increased AKT phosphorylation was noted after Ribociclib monotherapy in SNUC4 and DLD-1 cells. Alpelisib treatment alone significantly reduced pAKT levels in all cell lines, particularly in DLD-1. Similarly, levels of pS6 240/244 were reduced in all lines treated with Alpelisib and in Caco-2, LS1034, and SNUC4 cells treated with Ribociclib alone. However, the combination treatment had a more pronounced effect on pS6 240/244 reduction, especially in Caco-2 cells (mean difference = 0.8719; 95% CI = 0.5916 to 1.152; *p* = 0.0001) (Figure 3).

### 2.4. Effects of Combining Alpelisib and Ribociclib on Expression of Cell Cycle Proteins

A significant reduction in pRB levels was observed in Caco-2 (*PIK3CA/KRAS* wild-type) and SNUC4 (*PIK3CA* mutated) cells treated with the combination of Alpelisib and Ribociclib compared to controls. Additionally, E2F1 levels decreased in Caco-2 and LS1034 cells following combination treatment. Cyclin D1 expression increased in all cell lines treated with the combination, with statistical significance in SNUC4 (mean difference = −0.7462; 95% CI = −1.45 to −0.043; *p* = 0.0379) (Figure 3).

### 2.5. Effect of Combined Treatment with Alpelisib and Ribociclib on Apoptosis

There was a significant increase in pBcl-2 expression in Caco-2, LS1034, and DLD-1 cells treated with the combination of Alpelisib and Ribociclib, indicating enhanced apoptosis compared to single-agent treatments (Figure 3). However, cleaved caspase 9 was not detected in any of the cell lines after 72 h of treatment.

### 2.6. Anti-Tumour Activities of Alpelisib and Ribociclib and Their Combination in CAM Models of Colorectal Cancer

**Figure 4:**
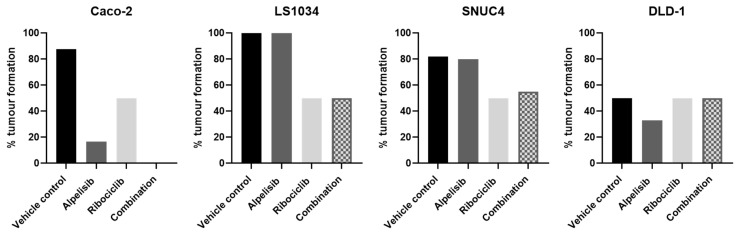
The percentage of macroscopically visible tumours formed in Caco-2 (*PIK3CA/KRAS* wild-type), LS1034 (*KRAS* mutated), SNUC4 (*PIK3CA* mutated), and DLD-1 (*KRAS/PIK3CA* mutated) CAM xenograft models. In total, 2 × 10^6^ Caco-2, LS1034, SNUC4, or DLD-1 cells were implanted into the CAM according to the assay schedule in Supplementary Figure S2 on day 7 of embryonic development. On day 10, tumours were treated with IC_50_ values of Alpelisib, Ribociclib, the combination of Alpelisib, and Ribociclib or DMSO vehicle control. On day 14, macroscopic tumour visibility was noted and expressed for each treatment as a percentage of the total number of viable eggs.

In CAM xenografts from Caco-2, LS1034, and SNUC4 cell lines, treatment with the combination of Alpelisib and Ribociclib resulted in fewer visible tumours formed at the study’s endpoint compared to vehicle-treated controls (Figure 4). Notably, in the Caco-2 group, the combination treatment delayed tumour development, with a statistically significant reduction in visible tumours compared to controls (*p* = 0.0022) (Supplementary Figure S1. In the DLD-1 group, 2/6 (33%) of combination-treated CAMs developed tumours, while the LS1034 group had 3/6 (50%), and the SNUC4 group had 5/9 (56%). In contrast, over 80% of DMSO-treated CAMs in all groups, except for DLD-1, had visible tumours by the study’s endpoint (Figure 4). However, failure of tumour formation in the DLD-1 CAMs was likely due to internal chick embryo factors rather than be treatment-related. Although there was some loss of xenografts throughout the experiment, this was relatively similar between the different treatment groups, indicating that the embryo death was not due to Alpelisib or Ribociclib toxicity.

### 2.7. Effects of Alpelisib and Ribociclib and Their Combination on Tumour Proliferation and Markers in CAM Models

**Figure 5:**
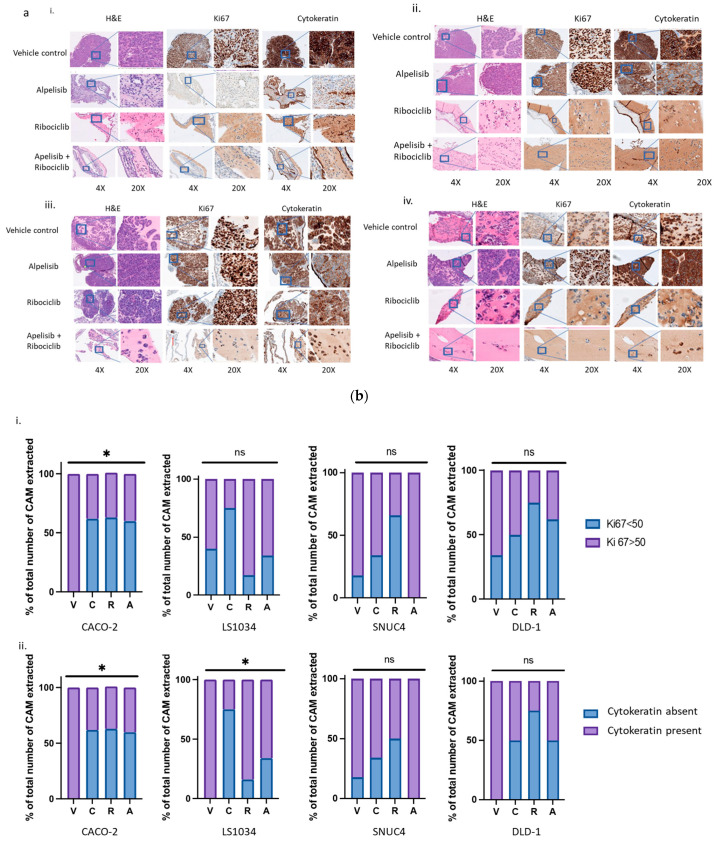
(**a**). Ki67 and cytokeratin immunohistochemistry in Caco-2 (**i**) (*PIK3CA/KRAS* wild-type), (**ii**) LS1034 (*KRAS* mutated), (**iii**) SNUC4 (*PIK3CA* mutated), and (**iv**) DLD-1 (*KRAS/PIK3CA* mutated) xenograft tumours. On day 14, the silicon ring and surrounding areas of CAM were excised, formalin-fixed and stained for Ki67 and cytokeratin. The stained sections were then imaged and analyzed using an Olympus Ckx41 microscope at 4× and 20× magnification. (**b**). Quantification of (**i**) Ki67 and (**ii**) cytokeratin staining in Caco-2 (*PIK3CA/KRAS* wild-type), LS1034 (*KRAS* mutated), SNUC4 (*PIK3CA* mutated), and DLD-1 (*KRAS/PIK3CA* mutated) xenograft tumours. For Ki67, data are expressed for each treatment as Ki67 > 50% or Ki67 < 50%. For cytokeratin, data are expressed as cytokeratin positive or negative for each treatment. Statistical significance was determined by Chi-square analysis. * *p* < 0.05, ns = not significant.

In the Caco-2 CAM model, 5/8 (62%) of tumours in the combination treatment group were negative for cytokeratin and had Ki67 staining less than 50%, similar to the Ribociclib group (62%) and the Alpelisib group (60%). By contrast, all tumours in the vehicle group were positive for cytokeratin and had Ki67 staining greater than 50%, with evidence of invading tumour cells (Figure 5). A similar pattern was observed in the DLD-1 CAM model, where 3/6 (50%) of combination-treated tumours showed no cytokeratin expression and less than 50% Ki67 staining. Interestingly, 100% of tumours in the DMSO group were positive for cytokeratin, though only 66% had Ki67 staining greater than 50%.

In the LS1034 model, the combination treatment resulted in 4/5 (75%) tumours being negative for cytokeratin and Ki67 staining less than 50%, compared to only 1/6 (16%) in the Ribociclib group. All DMSO-treated tumours were positive for cytokeratin, with some showing lower Ki67 staining due to embryo death (Figure 5).

In the SNUC4 model, 3/9 (34%) of combination-treated tumours were negative for cytokeratin, with less than 50% Ki67 staining. In the DMSO group, 9/11 (82%) of the tumours had Ki67 staining greater than 50%, with evidence of tumour cell invasion beyond the Matrigel (Figure 5).

## 3. Discussion

Despite advancements in cancer therapies, approximately 30% of colorectal cancer (CRC) patients still advance to Stage IV, with a 5-year overall survival (OS) rate of under 15% [6]. While the development of monoclonal antibodies, such as Cetuximab and Bevacizumab, has improved OS in metastatic CRC to over 24 months [7], most patients eventually experience relapse, and subsequent treatment lines generally have limited clinical impact. Numerous trials are underway to identify optimal treatment strategies to extend survival for Stage IV CRC patients whose disease progresses despite standard therapies. However, improvements in disease-free survival have not been significant. Resistance to anticancer treatments occurs through multiple mechanisms, making combination therapies a promising strategy to combat this challenge. Although CRCs often exhibit alterations in the PI3K pathway, monotherapies targeting this pathway have shown limited effectiveness [40,41].

Cyclin-dependent kinases (CDKs) play a crucial role in cell cycle regulation, and CDK4/6 inhibitors have been integrated into various clinical settings. However, when used alone, these inhibitors rarely lead to substantial tumour regression due to the emergence of resistance through diverse mechanisms [24,40,42]. Alpelisib, an orally bioavailable PI3K inhibitor that selectively targets the p110α isoform, has demonstrated sensitivity in PIK3CA-mutated cancers in preclinical models [34] and in Phase I trials involving patients with advanced solid tumours [43]. Alpelisib has been approved in combination with fulvestrant for treating *PIK3CA*-mutated breast cancer patients, based on the SOLAR-1 phase III trial results [44]. Ribociclib, a CDK4/6 inhibitor, has also shown efficacy in preclinical CRC models [45], and has been tested in combination with Alpelisib in early-phase breast cancer clinical trials [46]. This combination, aimed at inhibiting multiple pathways to prevent drug resistance in breast cancer, may also be applicable to CRC. Although histologically and molecularly distinct from breast adenocarcinoma, CRC shares certain fundamental features, particularly the role of PI3K and MAPK pathways in drug resistance [47]. The combined use of Alpelisib and Ribociclib could potentially counteract resistance by preventing RSK activation and MAPK pathway reactivation. However, there are limited studies exploring the potential of combining CDK4/6 and PI3K inhibitors in CRC patients. Our previous in vitro research demonstrated a strong synergistic effect between Palbociclib (CDK4/6 inhibitor) and Gedatolisib (PI3K inhibitor) in multiple CRC cell lines, regardless of mutation status [21]. Similarly, preclinical studies have shown that the combination of Alpelisib with Abemaciclib (CDK4/6 inhibitor) has synergistic effects in CRC models [48].

This study highlights that while both Ribociclib and Alpelisib induce cell cycle arrest and inhibit proliferation as monotherapies, their combination has a synergistic effect in CRC cell lines. Notably, the IC_50_ values for Alpelisib alone (0.313 µM to 1.54 µM) were lower than those reported for breast cancer cell lines [25]. Ribociclib exhibited higher IC_50_ values in DLD-1 (*PIK3CA/KRAS*-mutated) and Caco-2 (*PIK3CA/KRAS* wild-type) lines (IC_50_ >15 µM) compared to breast cancer models, though values for the SNUC4 (*PIK3CA*-mutated) and LS1034 (*KRAS*-mutated) lines were comparable [25]. This suggests that the presence of other mutations could make certain cell lines more responsive to the combination treatment.

A key observation was the synergistic anti-proliferative effect of combining Ribociclib and Alpelisib, which led to a significant reduction in pAKT, pS6, and more complete inhibition of the PI3K/AKT/mTOR pathway [49]. Reduced phosphorylated Rb levels, observed in all four cell lines, suggest that apoptosis was induced. Rb has been identified as a key predictive marker for CDK4/6 inhibitor efficacy in several studies [49,50,51]. The results indicate that pRB, pAkt, and pS6 may synergistically regulate cancer cell survival, and their simultaneous suppression could be critical for achieving optimal anti-tumour activity with the combination of Alpelisib and Ribociclib in CRC. Increased pBCL-2 levels in response to combination therapy in all cell lines further support apoptosis induction.

In the chick embryo chorioallantoic membrane (CAM) model, we observed a trend toward a reduction in tumour formation in xenografts treated with the Alpelisib–Ribociclib combination compared to single agents or vehicle controls, although due to xenograft loss, these results were not conclusive. Microscopic analysis also revealed reduced tumour growth with the combination therapy, as indicated by low cytokeratin and Ki67 staining.

Our study has some limitations. Only four cell lines were tested, and some important mutations, such as *KRAS* G12C, which may be more clinically relevant than G13D or A146T mutations, were not included. The drug combination produced synergistic effects in all four cell lines, regardless of *PIK3CA* or *KRAS* mutation status. However, the Caco-2 (*PIK3CA/KRAS* wild-type) cell line has a mutation in ERBB3, which may result in activation of the PI3K pathway. In the CAM experiments, the viability of the chick embryo was the main limiting factor, despite keeping the same supplier throughout the experiment, optimal incubation temperature and sterilization of the eggshell with 70% ethanol prior to starting the experiment. By the final day of the experiment (day 14), over 50% of xenografts were lost, likely due to embryo death rather than the treatments, though fungal infections also contributed to xenograft loss, in particular in the LS1034 xenografts where 13 out 35 eggs were lost throughout the experiment.

In summary, our study demonstrates that combining Alpelisib and Ribociclib results in a synergistic anti-tumour effect across colorectal cancer CRC cell lines with different mutational backgrounds. While both drugs individually affect cell viability, their combination proves more potent in suppressing cell proliferation and signalling pathways involved in cancer progression. The most significant impacts were seen in the reduction in pAKT, pS6, and cell cycle protein levels, particularly in DLD-1 and Caco-2 cells.

Additionally, the combination therapy enhances apoptosis markers and reduces tumour growth in in vivo CAM models. The results suggest that the combination of Alpelisib and Ribociclib could be a promising therapeutic strategy, particularly for CRC patients with *PIK3CA* or *KRAS* mutations. These findings support further investigations into the clinical potential of this drug combination to overcome resistance to monotherapies in CRC patients who have progressed after standard lines of treatment and improve patient outcomes.

## 4. Methods

### 4.1. Cell Lines and Drugs

Caco-2 (*PIK3CA/KRAS* wild-type) (RRID: CVCL_0025), LS1034 (*KRAS* A146T mutated) (RRID: CVCL_1382), and DLD-1 (*KRAS* G13D/*PIK3CA* E545K mutated) (RRID: CVCL_0248), were obtained from the American Type Culture Collection (ATCC, Manassas, VA, USA). The SNUC4 (*PIK3CA* E545G mutated) cell line (RRID: CVCL_5111) was acquired from the Korean Cell Line Bank (KCLB, Seoul, Republic of Korea). Cell lines were selected based on the presence of deleterious or potentially deleterious mutations in *PIK3CA* and/or *KRAS*. The authenticity of the cell lines was confirmed by Source BioScience (Nottingham, UK) using the AmpFISTR^®^ SGM Plus^®^ PCR amplification kit (Thermo Fisher Scientific, Waltham, MA, USA) before starting the study. Mutational status was checked through the Cancer Cell Line Encyclopedia (CCLE) [52] and verified by us using the MassARRAY system (Agena Bioscience™, Hamburg, Germany). Mycoplasma-free conditions were maintained throughout the experiments, with monthly testing using a Mycoalert detection kit (Lonza, Cambridge, MA, USA) LT 07-418). Ribociclib and Alpelisib were purchased from Selleckhem (Houston, TX, USA) and prepared as stock solutions in 100% dimethylsulfoxide (DMSO) at a concentration of 10 mM.

### 4.2. Acid Phosphatase Toxicity Assay

The acid phosphatase assay was used to evaluate the anti-proliferative response of the cell lines to Alpelisib and Ribociclib. On day 1, cells were plated in triplicate at a density of 1 × 10⁴ cells/mL into 96-well plates (100 µL per well) and incubated at 37 °C with 5% CO_2_ for 24 h to allow the cells to adhere. On day 2, cells were treated with 100 µL of serial 1:2 dilutions of the PI3K inhibitor Alpelisib (10 µM–0.08 µM) or the CDK4/6 inhibitor Ribociclib (20 µM–0.08 µM) or the DMSO control, and incubated for an additional 7 days. Following treatment, 100 µL of acid phosphatase substrate (para-Nitrophenylphosphate (pNPP) + 0.1 M sodium acetate, pH 5.5) was added, and absorbance was measured at 405 nm using a 96-well Victor™X3 Perkin Elmer multilabel plate reader. Each experiment was repeated 3–4 times. Inhibition of proliferation was calculated relative to untreated controls, and the half-maximal inhibitory concentration (IC_50_) for each drug was determined, which guided dosing for combination analysis.

### 4.3. SDS Polyacrylamide Gel Electrophoresis (SDS-PAGE) and Western Blotting

For SDS-PAGE, protein samples were mixed with a Laemmli buffer, and 10 µL of protein ladder (BenchMark™ Pre-stained Protein Ladder (Thermo Fisher Scientific, Waltham, MA, USA)) was loaded onto Bio-Rad Mini-PROTEAN^®^ TGX™ precast gels (Bio-Rad, Hercules, CA, USA). Electrophoresis was performed using a Bio-Rad Mini-PROTEAN^®^ II system. Western blotting was conducted by transferring proteins to membranes, which were then blocked in 5% BSA in TBS-T (0.1% Tween-20) and incubated overnight with primary antibodies at 4 °C. Membranes were incubated with horseradish peroxidase (HRP)-tagged secondary antibodies for 1 h at room temperature. Following washes in TBS-T, the membranes were immersed in enhanced chemiluminescence substrate (Novex™ECL HRP chemiluminescent reagent (Thermo Fisher Scientific, Waltham, MA, USA)) for 1 min before imaging using an Amersham imager (Cytiva, Marlborough, MA, USA). Antibodies and dilutions are detailed in Supplementary Table S1.

### 4.4. Chorioallantoic Membrane (CAM) Assay and Xenograft Generation

For the chorioallantoic membrane (CAM) assay, fertilized chicken eggs were obtained from St David’s Poultry (Newcastle West, Limerick, Ireland) and incubated at 37 °C from day 0. On day 3, a small window was cut in the egg and covered with a semi-permeable membrane. On day 8, a silicone ring was placed on the CAM, and 2 × 10^6^ cells mixed 1:1 with Matrigel (Corning^®^ Matrigel^®^ Basement Membrane Matrix (Corning, NY, USA)) were added inside the ring. On day 10, eggs were treated with Alpelisib, Ribociclib, their combination, or the DMSO (vehicle control) at the IC_50_ concentration (calculated from in vitro studies) in a total volume of 15 µL, applied directly to the xenografts. Ten eggs were treated per group. On day 14, embryos were sacrificed, and tumours were excised by cutting a 1 cm diameter around the silicon ring. The CAM study schema is provided in Supplementary Figure S1.

### 4.5. Xenograft Fixation and Immunohistochemical Analysis

Extracted xenografts were fixed overnight in formalin (Merck, Rahway, NJ, USA) and then transferred to 70% ethanol (Merck, Rahway, NJ, USA). Tumours were paraffin-embedded, sectioned, and immunohistochemically-stained for Ki67 (Invitrogen Ki-67 Recombinant Rabbit Monoclonal Antibody (SP6) (Thermo Fisher Scientific, Waltham, MA, USA)) and cytokeratin (MA51420Thermo Fisher Scientific, Waltham, MA, USA). Ki67 positivity was assessed as either greater than or less than 50% of cells. Stained sections were imaged using an Olympus Ckx41 microscope (Olympus Life Science, Waltham, MA, USA), and images were captured with QuPath software (version 0.5.1) (https://qupath.github.io accessed on 21 November 2024).

### 4.6. Statistical Analysis

GraphPad PRISM version 9.0 was used to calculate IC_50_ values for each drug and the combination index (CI) at the effective doses of ED50, ED75, and ED90, using the Chou-Talalay method (CalcuSyn, Biosoft, Ferguson, MO, USA) [39]. CI values were interpreted as synergistic (CI < 0.9), additive (0.9–1.0), or antagonistic (>1.1). Combination effects were assessed using one-way ANOVA (GraphPad PRISM version 9.0). Western blot band intensities were analyzed with ImageJ software (1.54b), and protein concentrations were derived using BSA standards. Data were presented as mean ± SEM and analyzed using ANOVA with Tukey’s multiple comparison tests. Chi-square analysis was used to evaluate the statistical significance of in vivo results. A *p*-value of <0.05 was considered statistically significant.

## Figures and Tables

**Table 1:** The inhibitory concentration 50 (IC_50_) values and subsequent combination drug doses used for Alpelisib and Ribociclib against colorectal cancer cell lines with various mutational backgrounds. The combination indexes (CI) at the effective doses of 50 (ED50), ED75, and ED90 for the combination of Alpelisib and Riboclicib are also shown. A CI < 0.9 is indicative of a synergistic effect (SY), between 0.9 and 1.0 is additive (AD), and >1.1 is antagonistic (AN). Each experiment was repeated three to four times.

Cell Line	Mutational Status	IC_50_ Alpelisib (A)	IC_50_ Ribociclib (R)	Drug Combination Doses (A + R)	CI@ED50	CI@ED75	CI@ED90
**Caco-2** **(ATCC HTB-37)**	*PIK3CA/KRAS* Wild-Type	1.547 µM	>15 µM	2 µM + 5 µM	0.36 ± 0.04 (SY)	0.45 ± 0.22 (SY)	0.60 ± 0.48 (SY)
**LS1034** **(ATCC CRL-2158)**	*KRAS* A146T	0.3134 µM	1.126 µM	400 nM + 2 µM	0.62 ± 0.33 (SY)	0.56 ± 0.25 (SY)	0.70 ± 0.33 (SY)
**SNUC4** **(KCLB 0000C4)**	*PIK3CA* E545G	0.4413 µM	0.259 µM	400 nM + 2 µM	1.08 ± 0.04 (AN)	0.56 ± 0.25 (SY)	0.23 ± 0.16 (SY)
**DLD-1** **(ATCC CCL-221)**	*KRAS* G13D *PIK3CA* E545K	1.307 µM	>15 µM	3 µM + 2 µM	0.06 ± 0.03 (SY)	0.03 ± 0.03 (SY)	0.03 ± 0.02 (SY)

## Data Availability

Data is contained within the article or Supplementary Material.

## Supplementary Materials

The following supporting information can be downloaded at: https://www.mdpi.com/article/10.3390/ijms252413264/s1.
