# Supplementary material for: Synergistic Effects of the Combination of Alpelisib (PI3K Inhibitor) and Ribociclib (CDK4/6 Inhibitor) in Preclinical Colorectal Cancer Models
Source: Int J Mol Sci. 2024 Dec 10;25(24):13264. doi: 10.3390/ijms252413264 (PMC11676898; doi:10.3390/ijms252413264)
Supplement: Supplementary file 1 [file ijms-25-13264-s001.zip › ijms-3357061-supplementary.pdf]

Supplementary Table S1: Primary antibodies used in the Western blot analyses for evaluation of the effect of Ribociclib, Alpelisib, and their combination in colorectal cancer cell lines.

No	Primary Antibody	Dilution	Source	Secondary Antibody
1	pMEK1/2 (S217/221) #9154	1:1000	Cell Signalling	Anti-rabbit Antibody
2	MEK1/2(L38C12)#4694	1:1000	Cell Signalling	Anti-mouse Antibody
3	Actin(BH10D10) #3700	1:1000	Cell Signalling	Anti-mouse Antibody
4	pAkt(Ser473)(D9E)XP#4060	1:1000	Cell Signalling	Anti-rabbit Antibody
5	Akt(pan)(C67E7)#4691	1:1000	Cell Signalling	Anti-rabbit Antibody
6	BCL-2 # 2872	1:1000	Cell Signalling	Anti-rabbit Antibody
7	pBCL-2 #2875	1:1000	Cell Signalling	Anti-rabbit Antibody
8	pRB S807/811#9308	1:1000	Cell Signalling	Anti-rabbit Antibody
9	RB (4H1) # 9309	1:1000	Cell Signalling	Anti-mouse Antibody
10	Cleaved caspase-9#9505	1:1000	Cell Signalling	Anti-rabbit Antibody
11	Caspase-9 #9502	1:1000	Cell Signalling	Anti-rabbit Antibody
12	E2F1 #3742	1:1000	Cell Signalling	Anti-rabbit Antibody
13	pS6 Ribosomal protein Sr240/244 #2215	1:1000	Cell Signalling	Anti-rabbit Antibody
14	S6 Ribosomal Protein (5G10) mAb #2217	1:1000	Cell Signalling	Anti-rabbit Antibody
15	Cyclin D1 # 2978	1:1000	Cell Signalling	Anti-rabbit Antibody

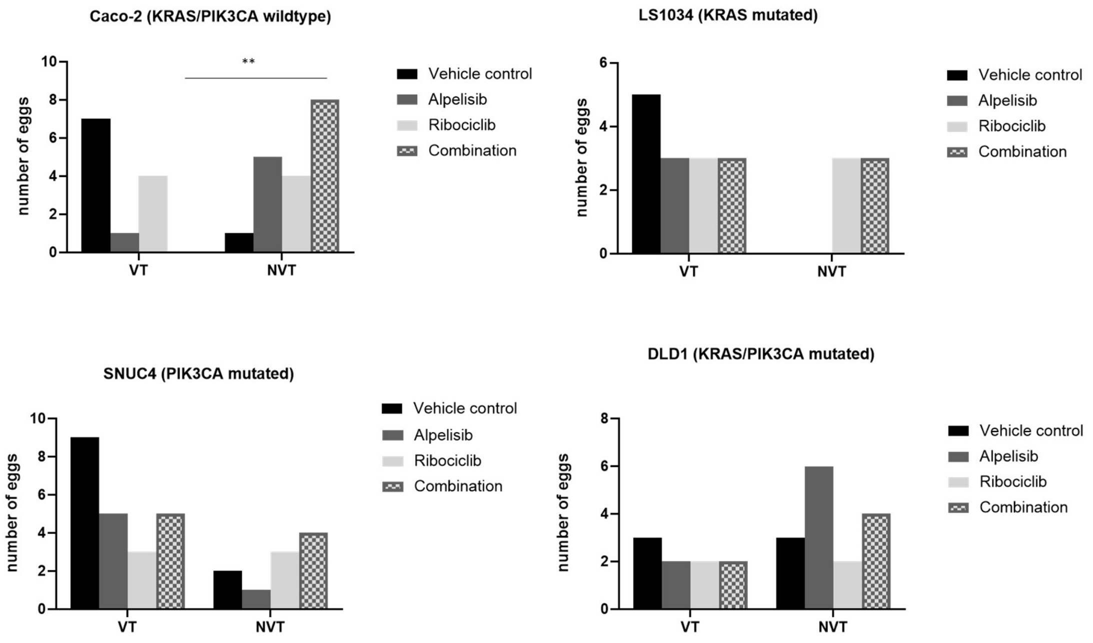

Supplementary Figure S1: Number of eggs with macroscopically visible tumours (VT) or non-visible tumours (NVT) in (a) Caco-2 (PIK3CA/KRAS wild-type) (b) LS1034 (KRAS mutated) (c) SNUC4 (PIK3CA mutated) and (d) DLD-1 (KRAS/PIK3CA mutated) CAM xenograft models. 2 x 10^6 Caco-2, LS1034, SNUC4 or DLD-1 cells were implanted into the CAM according to the assay schedule in Supplementary Figure S1 on day 7 of embryonic development. On day 10 tumours were treated with IC50 values of Alpelisib, Ribociclib, the combination of Apelisib and Ribociclib or DMSO vehicle control. On day 14, macroscopic tumour visibility was noted and expressed for each treatment as either visible tumour (VT) or non-visible (NVT). Statistical significance was determined by Chi square analysis. ** $p < 0.0022$.

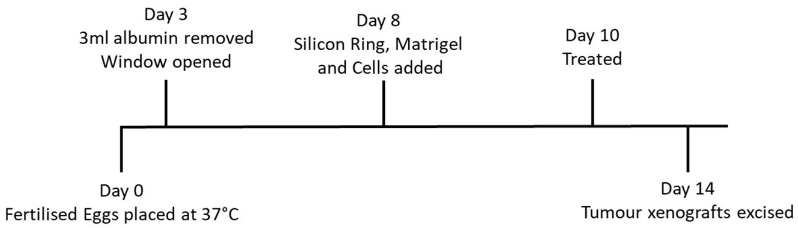

Supplementary Figure S2: CAM assay schedule
